# Adequate 25-hydroxyvitamin D levels are inversely associated with various cardiometabolic risk factors in Chinese children, especially obese children

**DOI:** 10.1136/bmjdrc-2019-000846

**Published:** 2020-02-17

**Authors:** Pei Xiao, Hongbo Dong, Haibo Li, Yinkun Yan, Hong Cheng, Junting Liu, Xiaoyuan Zhao, Dongqing Hou, Jie Mi

**Affiliations:** 1 Department of Epidemiology, Capital Institute of Pediatrics, Beijing, China; 2 Department of Non-communicable Disease Management, Beijing Children’s Hospital, Capital Medical University, National Center for Children’s Health, Beijing, China

**Keywords:** hyperglycemia, population-based interaction, cardiovascular disease risk, children

## Abstract

**Objective:**

Vitamin D deficiency has recently evolved as a major public health issue worldwide. But the relationship between vitamin D and cardiovascular health in children remains unclear. Accordingly, we aimed to examine the associations between 25-hydroxyvitamin D (25(OH)D) concentrations and cardiometabolic risk factors, and to assess the possible effect modification of obesity on the associations in a Chinese pediatric population.

**Research design and methods:**

A cross-sectional sample of 6091 children aged 6–18 years was obtained using a cluster sampling method. The 25(OH)D concentrations, and metabolic risk factors, including waist to height ratio, blood pressure, blood lipids, fasting blood glucose (FBG), and insulin were measured. Adjusted ORs and multiplicative or additive interaction were calculated to assess the associations and effect modification, respectively.

**Results:**

Triglycerides, FBG, insulin, and homeostasis model assessment of insulin resistance were inversely associated with 25(OH)D concentrations (p<0.05) in both sexes. The OR of hyperglycemia among individuals with insufficient vitamin D was higher than those with adequate vitamin D after adjusting for covariates (OR: 1.47; 95% CI 1.26 to 1.70). Moreover, girls with insufficient vitamin D had significantly higher odds for hypertension and high total cholesterol than those with adequate vitamin D, which was not observed in boys. Thirty-two percent (95% CI 14% to 51%) of the increased odds of hyperglycemia can be explained by the interaction between insufficient vitamin D and obesity.

**Conclusions:**

Vitamin D insufficiency is associated with increased odds of various cardiometabolic risk factors in Chinese children and has a synergistic effect on hyperglycemia with obesity.

**Significance of this study**

**What is already known about this subject?**

- The relationship between vitamin D and cardiometabolic risk factors has been debated.
- The bioavailability of vitamin D may be decreased due to excess storage in body fat compartments.

**What are the new findings?**

- Vitamin D insufficiency can increase the risk of hyperglycemia among children, and girls with insufficient vitamin D had significantly higher risk for hypertension and high total cholesterol.
- Vitamin D insufficiency has a synergistic effect on hyperglycemia with obesity.

**How might these results change the focus of research or clinical practice?**

- This study indicates that maintaining optimal levels of vitamin D in children, especially obese children, should be considered in future recommendations for cardiovascular disease prevention.

## Introduction

Vitamin D deficiency has recently evolved as a major public health issue worldwide that leads to rickets in children and osteomalacia and osteoporosis in adults.^1 2^ It is well understood that vitamin D can regulate calcium and phosphate homeostasis and bone metabolism. Vitamin D has also shown to play an important role in optimal function of various organs and tissues, including the cardiovascular system.^3^ The cellular effects of vitamin D which are mediated by ubiquitous vitamin D receptor were found in the heart, kidneys, brain and muscles.^4^ It has been demonstrated that vitamin D deficiency may be associated with several cardiovascular-related conditions such as metabolic syndrome, diabetes mellitus, hypertension and coronary artery disease.^5–9^ In adults, longitudinal associations between serum 25-hydroxyvitamin D (25(OH)D) concentration and cardiometabolic risk factors were observed in a number of cohort studies.^10–12^ However, there is insufficient evidence to support a causal link in children, and low serum 25(OH)D concentration may be a consequence rather than the cause of obesity and metabolic syndrome.^13^ The Chinese National Nutrition and Health Survey has reported that the prevalence of obesity and vitamin D deficiency in Chinese children was 16% and 53%, respectively.^14^ A multicenter survey among Chinese urban children found that the prevalence of hypercholesterolemia, hypertension, and hyperglycemia was 4%, 11%, and 23%, respectively.^15^ Both the vitamin D deficiency and cardiometabolic risk factors pose great health risks to children, and cardiometabolic risk factors in childhood may contribute to cardiovascular disease in adulthood.^16^ Nevertheless, few studies have addressed these associations between them in Chinese pediatric population.

Vitamin D, which has two biologically inactive parent compounds (cholecalciferol (vitamin D_3_) and ergocalciferol (vitamin D_2_)), is a fat-soluble and steroid-derived vitamin distributed in various tissues of the body.^13^ And fat sequestration of vitamin D in the expanded obese adipose tissue mass but further regional fat compartments may differ in metabolic function due to different expression of 25(OH)D metabolizing enzymes in subcutaneous and visceral adipose tissue in obese individuals.^13 17^ Bioavailability of vitamin D may be decreased due to excess storage in body fat compartments.^18^ Hence, adipose tissue may be a confounding or effect modification factor of the associations between vitamin D and cardiometabolic risk factors. Muscle is also a storage site for vitamin D metabolites by taking it up and releasing it into the circulation.^19^ Low 25(OH)D concentration, which represents the sum of 25-hydroxyergocalciferol and 25-hydroxycholecalciferol, was reported to be associated with cardiovascular risk factors in children.^5 6 8 9 20 21^ However, few studies have controlled for effect of body composition on the association between vitamin D deficiency and cardiovascular risk.

With a higher level of estrogens, the metabolism of vitamin D in girls may be different from that in boys. Moreover, cardiometabolic risk varies according to gender due to the distribution of subcutaneous, peripheral, and visceral fat.^22^ Thus, it is also important to examine such associations according to gender.^23^ In this study, we aimed to examine the gender-specific relationships between vitamin D and cardiometabolic risk factors (including abdominal obesity, hypertension, dyslipidemia, hyperglycemia, and insulin resistance), and to explore the effect modification of obesity on this association in a large Chinese pediatric population from China.

## Methods

### Participants

Data were obtained from a nationwide cross-sectional study conducted from January 2013 to December 2014, which was designed to examine cardiovascular health of school-age children in China. A stratified cluster sampling method was used to select a representative sample of children and adolescents aged 6–18 years in China. First, we chose three cities from north and two cities from south in China according to the characteristics of climate, economic development status and lifestyle habits. Then, two to three schools were randomly selected from each city, and all the students from the selected schools were invited to participate in questionnaire survey, anthropometric measurements and blood sample collection. Exclusion criteria included: (1) any condition, or use of any drug known to affect cardiovascular health; (2) non-removable objects (eg, deformity, fracture or prostheses); (3) pregnancy; (4) participation involving ionizing radiation in the past year; (5) absence from school; and (6) the inability to give informed consent. A total of 6976 children agreed to participate in the Dual-energy X-ray absorptiometry (DXA) scan and blood sample collection. For the present study, 6091 children (50.2% boys) who had valid data for 25(OH)D, cardiometabolic parameters and body composition were included for analysis.

### Data collection

Data collection was implemented in each school by trained staff. A standard self-administered questionnaire was pretested in a pilot study. It is a valid and reliable instrument to collect information on demographic characteristics (eg, age, sex and check month), personal and family medical history, and lifestyle factors (eg, drinking, smoking, physical activity (PA) and diet) among Chinese school-age children. Subjects answered questions mainly by themselves, or assisted by their parents/guardians when necessary (for those under 10 years old). Smoking was defined as having smoked or having smoking attempts in the past month. Alcohol drinking was defined as consumption of alcohol ≥18 g during the past month. Blood collection date was recorded and classified into spring (March to May), summer (June to August), autumn (September to November), and winter (December to February). Ideal PA was defined as daily frequency of moderate or vigorous PA more than 60 min. Dietary intake of vitamin D for the past 12 months was assessed by asking questions on frequency of milk/dairy products, fish/fish products, and eggs consumption. Subjects reported consuming food sources of vitamin D more than one time/day were considered having ideal dietary vitamin D intake.

### Physical examination

Height was measured to the nearest 0.1 cm by a stadiometer without shoes. Weight was measured to the nearest 0.1 kg using an electronic scale in fasting status with light indoor clothes. Weight and height were measured twice and the mean values were used for analysis. Body mass index (BMI) was calculated as weight (kg) divided by the square of height (m^2^). After resting for at least 15 min, blood pressure (BP) was measured three times with 1–2 min intervals (OMRON HEM-7012, Omron, Kyoto, Japan) in a sitting position from the right arm using a suitable cuff size based on the arm circumference. The average of the last two readings was used for analysis. Waist circumference was measured in the mid-way between the lowest rib and the superior border of the iliac crest with an inelastic measuring tape at the end of normal expiration to the nearest 0.1 cm. Waist to height ratio (WHtR) was calculated as waist circumference (cm) divided by the height (cm).

Body composition (fat mass, muscle mass, abdominal visceral adipose tissue and subcutaneous adipose tissue mass) was measured by the whole-body DXA using Hologic Discovery (A, W and Wi) fan-beam densitometers (Hologic, Bedford, Massachusetts, USA). The coefficient of variation (CV) was used as a quality control procedure. CV% of A, W and Wi was 0.471%, 0.302% and 0.358%, respectively. Fat mass percentage (FMP) was calculated as total fat mass (kg) divided by the weight (kg). Fat mass index (FMI) was calculated as total fat mass (kg) divided by the square of height (m^2^), and muscle mass index (MMI) was calculated as total muscle mass (kg) divided by the square of height (m^2^).

### Laboratory analysis

Venous blood samples were collected in tubes containing liquid EDTA after a 12-hour overnight fast and centrifuged at 4°C. Specimens collected in each center were shipped on dry ice by air to the central clinical laboratory of Capital Institute of Pediatrics in Beijing. The specimens were stored at −80℃ until analysis. Plasma glucose (enzyme hexokinase method) and lipids (enzymatic methods) were directly measured using the Hitachi 7080 biochemistry autoanalyzer (Hitachi, Tokyo, Japan). Plasma insulin was determined by a radioimmunoassay (Cisbio Bioassays, Codolet, France), which had no cross-reactivity to proinsulin. Plasma 25(OH)D concentrations were determined by using DiaSorin 25OH Vitamin D total assay (DiaSorin, Stillwater, MN, USA) on an automated chemiluminescent platform. All the intra-assay and interassay coefficients of variation were <5% and<10%, respectively.

### Definitions and diagnostic criteria

Weight status was classified as normal and obesity (including overweight) according to sex and age-specific BMI cut-off values recommended by the International Obesity Task Force.^24^ Abdominal obesity was defined as WHtR greater than 0.5.^25^ Individuals with average systolic blood pressure (SBP) and/or diastolic blood pressure (DBP) ≥95th sex, age and height-specific percentile for Chinese children and adolescents, or taking antihypertensive drugs, were classified as hypertension.^26^ Dyslipidemia was defined by the sex and age-specific lipoprotein cut-points based on Chinese children and adolescents^27^ or taking antihyperlipidemia medications. Fasting hyperglycemia was defined as fasting blood glucose (FBG) ≥5.6 mmol/L or taking antihyperglycemia medications.^28^ Insulin resistance was estimated using the homeostasis model assessment of insulin resistance (HOMA-IR) by the following formula: fasting insulin (mU/L)×FBG (mmol/L)/22.5. Insulin resistance was defined by the WHO as values in the highest quartile of the HOMA-IR.^29^

The status of 25(OH)D concentrations was classified as deficiency (<30 nmol/L), inadequacy (30 to <50 nmol/L), and adequacy (≥50 nmol/L) according to the report released in 2010 by the Institute of Medicine.^30^ For logistic regression and interaction analysis, vitamin D status was also dichotomized into adequacy or insufficiency with a threshold of 50 nmol/L.

### Statistical analyses

R software (V.3.4.0, www.cran.r-project.org) was used for all calculations and analyses. Data were expressed as mean (SDs) for normally distributed variables, geometric mean (95% CIs) for skewed variables, and frequency (%) for categorical variables. To compare the differences in characteristics among weight status groups in both sexes, the Pearson χ^2^ test was applied for categorical variables, and the analysis of variance and Kruskal-Wallis H test were chosen for normally distributed and skewed variables, respectively. In the multivariable analysis, variables associated with both vitamin D levels and cardiometabolic risk factors were adjusted as confounders, including sex (not for sex-stratified analysis), age, season of blood collection, geographical location, smoking, drinking, diet habitats, PA, BMI, FMP, and MMI.

Linear associations between 25(OH)D concentrations and cardiometabolic parameters were determined by partial correlation analysis and we adjusted FMI instead of FMP in the sensitivity analysis. We used restricted cubic splines in generalized additive models to further explore the non-linear associations between them. Multivariate adjusted means of cardiometabolic parameters in different vitamin D status groups were calculated using general linear model. P value for multiple group statistical comparison was corrected using Dunnett method. Then the trend in mean values across groups was tested by treating vitamin D status groups as a continuous variable. Multiple logistic regression models were applied to examine associations between vitamin D status and cardiometabolic abnormalities. Multivariate adjusted ORs and 95% CIs were calculated for the presence of cardiometabolic risk factors in each vitamin D status category. ORs for vitamin D deficiency, inadequacy, or insufficiency (deficiency and inadequacy) in relation to adequacy (referent category) were compared.

To explore the potential effect modification of obesity on the associations between vitamin D and cardiometabolic risk factors, we conducted interaction analyses on both multiplicative and additive scales. A cross-product interaction term was included in the logistic regression model to assess multiplicative interaction. The relative excess risk due to interaction (RERI) and attributable proportion (AP) due to interaction were calculated to assess additive interaction.^31^ In the absence of interaction, both RERI and AP are equal to 0. In addition to BMI, we also used WHtR to measure obesity in the interaction analysis. A two-tailed p≤0.05 was considered significant in all analyses.

## Results

### Characteristics of participants

This study included 6091 participants (3057 boys and 3034 girls). The mean age of participants was 11.9±3.7 years. The overall mean of plasma 25(OH)D concentrations was 39.0±15.1 nmol/L, which was significantly higher in boys than in girls (p<0.001). The prevalence of vitamin D insufficiency was 78.4% (30.3% for deficiency) among study population, and a higher prevalence of insufficiency was significant in girls rather than in boys (81.6 vs 75.1%, p<0.001). Demographic characteristics and cardiometabolic risk factors of participants stratified by sex and vitamin D status were presented in table 1.

**Table 1:** Characteristics of participants stratified by sex and vitamin D status*

Characteristics†	Boys (n=3057)				Girls (n=3034)			
	Adequacy	Inadequacy	Deficiency	P value	Adequacy	Inadequacy	Deficiency	P value
n	760	1487	810		557	1441	1036	
Age, mean (SD), years	11.66 (4.04)	12.37 (3.71)	12.74 (3.34)	<0.001	11.43 (4.05)	12.75 (3.85)	13.16 (3.42)	<0.001
25(OH)D, mean (SD), nmol/L	61.13 (10.14)	39.46 (5.55)	23.00 (5.07)	<0.001	60.66 (10.00)	39.11 (5.56)	22.63 (5.05)	<0.001
Season of blood collection, n (%)				<0.001				<0.001
Spring	167 (22.0)	505 (34.0)	233 (28.8)		111 (19.9)	387 (26.9)	292 (28.2)	
Summer	174 (22.9)	154 (10.4)	24 (3.0)		158 (28.4)	166 (11.5)	47 (4.5)	
Autumn	349 (45.9)	691 (46.5)	452 (55.8)		240 (43.1)	756 (52.5)	571 (55.1)	
Winter	70 (9.2)	137 (9.2)	101 (12.5)		48 (8.6)	132 (9.2)	126 (12.2)	
North, n (%)	523 (68.8)	1012 (68.1)	567 (70.0)	0.630	373 (67.0)	895 (62.1)	742 (71.6)	<0.001
Smoking, n (%)	74 (9.7)	140 (9.4)	92 (11.4)	0.320	29 (5.2)	73 (5.1)	50 (4.8)	0.938
Drinking, n (%)	162 (21.3)	315 (21.2)	191 (23.6)	0.380	96 (17.2)	259 (18.0)	180 (17.4)	0.894
Ideal PA, n (%)	243 (32.0)	480 (32.3)	222 (27.4)	0.042	104 (18.7)	279 (19.4)	211 (20.4)	0.690
Ideal dietary vitamin D intake, n (%)	60 (7.9)	119 (8.0)	52 (6.4)	0.359	43 (7.7)	82 (5.7)	60 (5.8)	0.207
BMI, mean (SD), kg/m^2^	19.04 (3.95)	20.22 (4.47)	20.32 (4.72)	<0.001	18.30 (3.61)	19.34 (3.79)	19.52 (3.83)	<0.001
FMP, geometric mean (95% CI), %	25.4 (24.9 to 25.9)	26.9 (26.5 to 27.3)	26.3 (25.8 to 26.8)	<0.001	31.2 (30.7 to 31.7)	32.6 (32.2 to 32.8)	32.1 (31.7 to 32.5)	<0.001
FMI, geometric mean (95% CI), kg/m^2^	4.76 (4.63 to 4.89)	5.31 (5.20 to 5.43)	5.20 (5.04 to 5.36)	<0.001	5.62 (5.47 to 5.78)	6.22 (6.11 to 6.33)	6.16 (6.03 to 6.29)	<0.001
MMI, geometric mean (95% CI), kg/m^2^	13.1 (12.9 to 13.3)	13.5 (13.4 to 13.6)	13.7 (13.5 to 13.8)	<0.001	11.7 (11.5 to 11.8)	12.1 (12.0 to 12.2)	12.2 (12.1 to 12.4)	<0.001
Obesity (including overweight), n (%)	176 (23.2)	483 (32.5)	239 (29.5)	<0.001	88 (15.8)	279 (19.4)	195 (18.8)	0.176
Abdominal obesity, n (%)	119 (15.7)	339 (22.8)	173 (21.4)	<0.001	46 (8.3)	162 (11.2)	116 (11.2)	0.123
Hypertension, n (%)	161 (21.2)	361 (24.3)	184 (22.7)	0.247	86 (15.4)	337 (23.4)	246 (23.7)	<0.001
High TC, n (%)	10 (1.3)	41 (2.8)	21 (2.6)	0.090	15 (2.7)	74 (5.1)	50 (4.8)	0.058
High LDL-C, n (%)	16 (2.1)	51 (3.4)	31 (3.8)	0.122	12 (2.2)	64 (4.4)	35 (3.4)	0.043
High HDL-C, n (%)	97 (12.8)	214 (14.4)	119 (14.7)	0.482	7 (1.3)	9 (0.6)	5 (0.5)	0.188
High TG, n (%)	28 (3.7)	96 (6.5)	72 (8.9)	<0.001	9 (1.6)	25 (1.7)	35 (3.4)	0.013
Hyperglycemia, n (%)	277 (36.4)	585 (39.3)	318 (39.3)	0.372	115 (20.6)	385 (26.7)	324 (31.3)	<0.001
Insulin resistance, n (%)	149 (19.6)	338 (22.7)	199 (24.6)	0.058	116 (20.8)	327 (22.7)	259 (25.0)	0.146

Data were presented as mean (SD), geometric mean (95% CI), or n (%).

*Vitamin D status was classified by the Institute of Medicine (IOM) recommendation: deficiency, <30 nmol/L; inadequacy, 30 to <50 nmol/L; adequacy, ≥50 nmol/L.^20^

†The diagnostic criteria are as follows: The weight status was classified as normal and obesity (including overweight) according to the International Obesity Task Force (IOTF) criteria^14^; Abdominal obesity was defined as waist to height ratio ≥0.5^15^; Hypertension was classified by the 95th sex, age and height-specific blood pressure cut-points of Chinese standard^16^; Abnormal blood lipid levels were classified by the age and sex-specific lipoprotein cut-points of Chinese children^17^; Hyperglycemia was defined as fasting blood glucose ≥5.6 mmol/L^18^; Insulin resistance was defined by the WHO as values in the highest quartile of the homeostasis model assessment of insulin resistance (HOMA-IR).^19^

BMI, body mass index; FMI, fat mass index; FMP, fat mass percentage; HDL-C, high-density lipoprotein-cholesterol; LDL-C, low-density lipoprotein-cholesterol; MMI, muscle mass index; 25(OH)D, 25-hydroxyvitamin D; PA, physical activity; TC, total cholesterol; TG, triglyceride.

### Associations between plasma 25(OH)D concentrations and cardiometabolic parameters

After adjusting for age, sex, season of blood collection, geographical location, smoking, drinking, PA, dietary vitamin D intake, BMI, FMP, and MMI, the partial correlation analysis showed that plasma 25(OH)D concentration was inversely associated with SBP, triglyceride (TG), FBG, insulin, and HOMA-IR; while it was positively associated with WHtR and high-density lipoprotein-cholesterol (HDL-C) (table 2). However, the sex-stratified analysis showed that plasma 25(OH)D concentration was positively associated with DBP in boys but negatively correlated with DBP in girls. In the sensitivity analysis, the partial correlations did not significantly change after adjusting for FMI instead of FMP (online supplementary table 1). Interestingly, plasma 25(OH)D concentration showed inverse U-shaped associations with total cholesterol (TC), low-density lipoprotein-cholesterol (LDL-C), and HDL-C in both sexes (online supplementary figure 1).

10.1136/bmjdrc-2019-000846.supp1 Supplementary data

**Table 2:** Partial correlations between 25(OH)D and cardiometabolic parameters*

Cardiometabolic parameters	All (n=6091)		Boys (n=3057)		Girls (n=3034)	
	r	P value	r	P value	r	P value
WHtR	0.035	0.006	0.044	0.014	0.029	0.109
SBP	−0.035	0.007	0.022	0.227	−0.107	<0.001
DBP	−0.012	0.356	0.038	0.035	−0.068	<0.001
TC	0.006	0.621	−0.001	0.948	0.012	0.496
LDL-C	0.010	0.436	−0.002	0.933	0.018	0.319
HDL-C	0.043	<0.001	0.052	0.004	0.035	0.055
TG**†**	−0.044	<0.001	−0.046	0.011	−0.041	0.024
FBG**†**	−0.035	0.007	−0.027	0.033	−0.041	0.025
Insulin**†**	−0.064	<0.001	−0.066	<0.001	−0.064	<0.001
HOMA-IR**†**	−0.070	<0.001	−0.070	<0.001	−0.072	<0.001

*Model adjusted for age, sex (not for adjusted in stratified analysis), season of blood collection, geographical location, smoking, drinking, physical activity, dietary vitamin D intake, body mass index (BMI), fat mass percentage (FMP), and muscle mass index (MMI).

†Log transformed.

DBP, diastolic blood pressure; FBG, fasting blood glucose; HDL-C, high-density lipoprotein-cholesterol; HOMA-IR, homeostasis model assessment for insulin resistance; LDL-C, low-density lipoprotein-cholesterol; 25(OH)D, 25-hydroxyvitamin D; SBP, systolic blood pressure; TC, total cholesterol; TG, triglyceride; WHtR, waist to height ratio.

**Figure 1:**
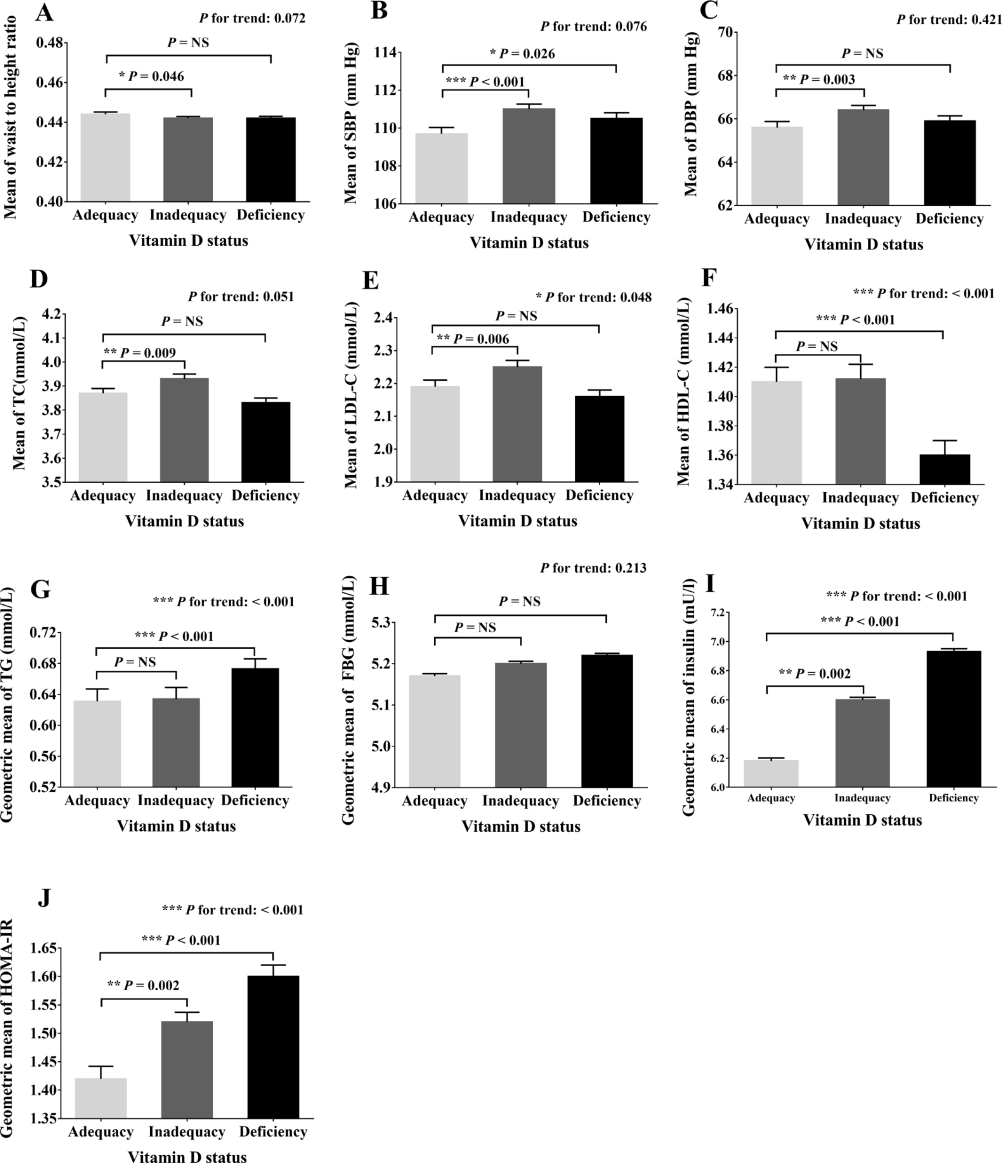
Multivariate adjusted means or geometric means of cardiometabolic parameters across different vitamin D status groups. (A)–(J) stand for the adjusted means (or geometric means) of waist to height ratio (WHtR), systolic blood pressure (SBP), diastolic blood pressure (DBP), total cholesterol (TC), low-density lipoprotein-cholesterol (LDL-C), high-density lipoprotein-cholesterol (HDL-C), triglyceride (TG), fasting blood glucose (FBG), insulin, and homeostasis model assessment of insulin resistance (HOMA-IR), respectively. Means (or geometric means) are adjusted for age, sex, season of blood collection, geographical location, smoking, drinking, physical activity, dietary vitamin D intake, body mass index (BMI), fat mass percentage (FMP), and muscle mass index (MMI). Error bars stand for SE. *0.01≤p<0.05; **0.001≤p<0.01; ***p<0.001. NS, not significant.

Multivariate adjusted means or geometric means of cardiometabolic parameters across vitamin D status groups were presented in figure 1. Participants with vitamin D deficiency or inadequacy had higher levels of SBP, insulin, and HOMA-IR than those with vitamin D adequacy (p<0.05). Significantly increasing trends (p for trend <0.001) of insulin and HOMA-IR levels were observed across vitamin D status groups after multivariate adjustment. Likewise, the mean levels of TC and LDL-C in the vitamin D inadequacy category and levels of TG in the vitamin D deficiency category were significantly higher than those in the vitamin D adequacy category (p<0.05). In the sex-stratified analysis, the differences in SBP were not statistically significant across vitamin D groups in boys, while increasing levels of SBP and DBP (p for trend <0.001) were shown as vitamin D levels decreased in girls (online supplementary figures 2 and 3).

### Associations between vitamin D status and cardiometabolic abnormalities

ORs of cardiometabolic abnormalities across different vitamin D status groups were examined using a multiple logistic regression analysis (table 3). After multivariate adjustment, a significantly higher odds of hyperglycemia was noticed in participants with deficient and inadequate vitamin D than those with adequate vitamin D. Additionally, in comparison to children with adequate vitamin D, odds of high TC, LDL-C and TG were significantly higher in children with insufficient vitamin D level. Plasma 25(OH)D concentrations were negatively associated with the prevalence of high TG and hyperglycemia in multivariate adjusted (p for trend <0.001) logistic regression analysis. The sex-stratified analysis showed that girls with insufficient vitamin D had significantly higher odds of hypertension than those with adequate vitamin D, which was not observed in boys.

**Table 3:** Multivariate adjusted ORs (95% CIs)* of cardiometabolic risk factors according to vitamin D status

Cardiometabolic risk factors†	Vitamin D status‡				
	Adequacy	Inadequacy	Deficiency	P for trend	Insufficiency (inadequacy and deficiency)
Overall					
Abdominal obesity	1.00	0.88 (0.69 to 1.11)	0.94 (0.72 to 1.23)	0.458	0.86 (0.68 to 1.08)
Hypertension	1.00	1.23 (1.01 to 1.50)§	1.08 (0.87 to 1.34)	0.823	1.18 (0.97 to 1.42)
High TC	1.00	1.67 (1.07 to 2.62)§	1.55 (0.96 to 2.51)	0.052	1.80 (1.16 to 2.80)§
High LDL-C	1.00	1.56 (1.02 to 2.40)§	1.42 (0.89 to 2.27)	0.209	1.56 (1.02 to 2.38)§
Low HDL-C	1.00	1.19 (0.91 to 1.56)	1.36 (0.99 to 1.85)	0.459	1.10 (0.84 to 1.43)
High TG	1.00	1.21 (0.81 to 1.80)	2.05 (1.35 to 3.10)§	<0.001	1.64 (1.12 to 2.41)§
Hyperglycemia	1.00	1.17 (1.01 to 1.36)§	1.24 (1.05 to 1.46)§	<0.001	1.47 (1.26 to 1.70)§
Insulin resistance	1.00	1.12 (0.94 to 1.32)	1.19 (0.98 to 1.43)	0.915	1.03 (0.87 to 1.21)
Boys					
Abdominal obesity	1.00	1.11 (0.76 to 1.64)	0.78 (0.49 to 1.23)	0.227	1.01 (0.70 to 1.47)
Hypertension	1.00	1.13 (0.88 to 1.45)	0.89 (0.67 to 1.18)	0.162	1.04 (0.82 to 1.32)
High TC	1.00	1.46 (0.92 to 2.32)	1.61 (0.94 to 2.75)	0.095	1.50 (0.96 to 2.34)
High LDL-C	1.00	1.19 (0.70 to 2.03)	1.42 (0.78 to 2.59)	0.247	1.25 (0.75 to 2.10)
Low HDL-C	1.00	1.00 (0.52 to 1.90)	1.05 (0.49 to 2.23)	0.902	1.01 (0.54 to 1.88)
High TG	1.00	1.23 (0.86 to 1.76)	1.85 (1.23 to 2.76)§	0.002	1.38 (0.98 to 1.94)
Hyperglycemia	1.00	1.23 (1.01 to 1.51)§	1.45 (1.14 to 1.83)§	0.002	1.29 (1.07 to 1.57)§
Insulin resistance	1.00	1.08 (0.86 to 1.37)	1.10 (0.84 to 1.44)	0.515	1.09 (0.87 to 1.36)
Girls					
Abdominal obesity	1.00	1.15 (0.78 to 1.69)	1.22 (0.80 to 1.86)	0.389	1.17 (0.81 to 1.70)
Hypertension	1.00	1.54 (1.10 to 2.16)§	1.66 (1.16 to 2.39)§	0.018	1.58 (1.14 to 2.19)§
High TC	1.00	1.88 (1.17 to 3.01)§	1.54 (0.91 to 2.59)	0.317	1.77 (1.12 to 2.82)§
High LDL-C	1.00	1.85 (1.10 to 3.11)§	1.29 (0.72 to 2.31)	0.902	1.66 (0.99 to 2.78)
Low HDL-C	1.00	1.62 (0.75 to 3.50)	2.25 (0.92 to 5.53)	0.076	1.75 (0.83 to 3.70)
High TG	1.00	1.15 (0.77 to 1.71)	2.10 (1.37 to 3.24)§	<0.001	1.39 (0.95 to 2.02)
Hyperglycemia	1.00	1.65 (1.28 to 2.13)§	2.16 (1.64 to 2.85)§	<0.001	1.81 (1.41 to 2.31)§
Insulin resistance	1.00	0.97 (0.75 to 1.26)	0.95 (0.72 to 1.26)	0.705	0.96 (0.75 to 1.24)

*The analysis was adjusted for age, sex (not for adjusted in stratified analysis), season of blood collection, geographical location, smoking, drinking, physical activity, dietary vitamin D intake, body mass index (BMI), fat mass percentage (FMP), and muscle mass index (MMI).

†The diagnostic criteria are as follows: Abdominal obesity was defined as waist to height ratio ≥0.5^15^; Hypertension was classified by the 95th sex, age and height-specific blood pressure cut-points of Chinese standard^16^; Abnormal blood lipids levels were classified by the age and sex-specific lipoprotein cut-points of Chinese children^17^; Hyperglycemia was defined as fasting blood glucose ≥5.6 mmol/L^18^; Insulin Rresistance was defined by the World Health OrganizationWHO as values in the highest quartile of the homeostasis model assessment of insulin resistance (HOMA-IR).^19^

‡Vitamin D status was classified by the Institute of Medicine (IOM) recommendation: deficiency, <30 nmol/L; inadequacy, 30 to <50 nmol/L; adequacy, ≥50 nmol/L.^20^

§Significantly different from the referent category, vitamin D adequacy group.

HDL-C, high-density lipoprotein-cholesterol; LDL-C, low-density lipoprotein-cholesterol; TC, total cholesterol; TG, triglyceride.

### Joint effects of vitamin D insufficiency and obesity (including overweight) on cardiometabolic abnormalities

Table 4 presented the joint effects of vitamin D insufficiency and obesity (including overweight) on cardiometabolic risk factors after adjusting for confounders. Obese individuals with vitamin D insufficiency had a significantly increased odds of hyperglycemia compared with normal weight individuals with adequate vitamin D (OR: 1.73, 95% CI 1.39 to 2.15). Significant multiplicative and additive interactions of vitamin D insufficiency and obesity on hyperglycemia were both observed. The odds of hyperglycemia in obese individuals with vitamin D insufficiency was 0.56 (95% CI 0.22 to 0.90) more than that if there was no interaction between obesity and vitamin D insufficiency. The proportion of joint effect on hyperglycemia due to interaction was 32% (95% CI 0.14 to 0.51). However, there was no evidence of interaction of insufficient 25(OH)D with abdominal obesity on these cardiometabolic abnormalities (online supplementary table 2).

**Table 4:** Multivariate adjusted ORs of cardiometabolic risk factors according to vitamin D status and weight status groups*

Cardiometabolic risk factors †	Weight status ‡	Vitamin D status §	OR (95% CI)	P for interaction¶	Measures of additive interaction	
					RERI (95% CI)	AP (95% CI)
Abdominal obesity	Normal	Adequacy	1.00	0.583	3.55 (−5.70 to 12.79)	0.12 (−0.18 to 0.41)
		Insufficiency	1.32 (0.89 to 1.97)			
	Obesity	Adequacy	26.23 (16.22 to 42.42) **			
		Insufficiency	30.10 (20.12 to 45.04) **			
Hypertension	Normal	Adequacy	1.00	0.146	−0.44 (−1.27 to 0.37)	−0.20 (−0.57 to 0.17)
		Insufficiency	1.19 (0.95 to 1.48)			
	Obesity	Adequacy	2.49 (1.73 to 3.60) **			
		Insufficiency	2.23 (1.70 to 2.93) **			
High TC	Normal	Adequacy	1.00	0.805	−0.07 (−0.69 to 0.55)	−0.06 (−0.57 to 0.45)
		Insufficiency	1.56 (1.08 to 2.26) **			
	Obesity	Adequacy	0.70 (0.36 to 1.35)			
		Insufficiency	1.19 (0.76 to 1.86)			
High LDL-C	Normal	Adequacy	1.00	0.540	0.43 (−0.4 to 1.26)	0.23 (−0.21 to 0.67)
		Insufficiency	1.35 (0.87 to 2.09)			
	Obesity	Adequacy	1.12 (0.54 to 2.29)			
		Insufficiency	1.90 (1.15 to 3.13) **			
Low HDL-C	Normal	Adequacy	1.00	0.873	0.06 (−0.88 to 1.01)	0.03 (−0.37 to 0.43)
		Insufficiency	1.99 (1.01 to 3.94) **			
	Obesity	Adequacy	5.03 (2.09 to 12.09) **			
		Insufficiency	3.73 (1.72 to 8.13) **			
High TG	Normal	Adequacy	1.00	0.074	−0.37 (−1.58 to 0.83)	−0.11 (−0.46 to 0.24)
		Insufficiency	1.70 (1.21 to 2.39) **			
	Obesity	Adequacy	3.14 (1.98 to 4.97) **			
		Insufficiency	3.47 (2.37 to 5.08) **			
Hyperglycemia	Normal	Adequacy	1.00	0.011	0.56 (0.22 to 0.90)	0.32 (0.14 to 0.51)
		Insufficiency	1.33 (1.12 to 1.57) **			
	Obesity	Adequacy	0.84 (0.61 to 1.16)			
		Insufficiency	1.73 (1.39 to 2.15) **			
Insulin resistance	Normal	Adequacy	1.00	0.606	−0.11 (−0.62 to 0.39)	−0.07 (−0.41 to 0.26)
		Insufficiency	1.06 (0.87 to 1.29)			
	Obesity	Adequacy	1.57 (1.12 to 2.19) **			
		Insufficiency	1.51 (1.19 to 1.93) **			

*The analysis was adjusted for age, sex, season of blood collection, geographical location, smoking, drinking, physical activity, dietary vitamin D intake, body mass index (BMI), fat mass percentage (FMP), and muscle mass index (MMI).

†The diagnostic criteria are as follows: Abdominal obesity was defined as waist to height ratio ≥0.5^15^; Hypertension was classified by the 95th sex, age and and height-specific blood pressure cut-points of Chinese standard^16^; Abnormal blood lipid levels were classified by the age and sex-specific lipoprotein cut-points of Chinese children^17^; Hyperglycemia was defined as fasting blood glucose ≥5.6 mmol/L^18^; Insulin resistance was defined by the WHO as values in the highest quartile of the homeostasis model assessment of insulin resistance (HOMA-IR).^19^

‡The weight status was classified as normal and obesity (including overweight) according to the International Obesity Task Force (IOTF) criteria.^14^

§The vitamin D status was classified as sufficiency (≥50 nmol/L) and insufficiency (<50 nmol/L) according to the Institute of Medicine (IOM) recommendation.^20^

¶A cross-product interaction term was included in the logistic regression model to assess multiplicative interaction.

**Significantly different from the referent category, vitamin D sufficiency and normal weight group.

AP, attributable proportion; HDL-C, high-density lipoprotein-cholesterol; LDL-C, low-density lipoprotein-cholesterol; RERI, relative excess risk due to interaction; TC, total cholesterol; TG, triglyceride.

## Discussion

In this nationwide cross-sectional study investigating associations between plasma 25(OH)D concentrations and cardiometabolic risk factors, we reported that 25(OH)D concentration was inversely related to TG, FBG, insulin, and HOMA-IR in both genders, but only positively associated with WHtR, DBP and HDL-C in boys and negatively associated with SBP and DBP in girls. In addition, individuals with vitamin D insufficiency had significantly increased risk of hyperglycemia in both genders as well as hypertension and high TC in girls. Interestingly, a significant effect modification of vitamin D insufficiency and obesity on hyperglycemia was observed, and 32% (95% CI 14% to 51%) of the increased odds of hyperglycemia can be explained by the interaction.

Although several epidemiological studies have observed that vitamin D deficiency was associated with obesity in children, the causality of association has remained uncertain.^5 8 14 32–36^ Low levels of 25(OH)D concentration may work as a marker rather than a cause of obesity.^37^ On the basis of a bidirectional genetic approach that limits confounding, a Mendelian randomization study suggested that a higher BMI leads to lower 25(OH)D, while any effects of lower 25(OH)D increasing BMI are likely to be small.^38^ Thus, it is most likely that high storage capacity of vitamin D in adipose tissue leads to low circulatory 25(OH)D levels.^18^

Vitamin D deficiency during childhood is known to cause growth retardation and skeletal deformities. However, the fact that hydroxylase enzymes and vitamin D receptor (VDR) are expressed in many different cell types indicates that vitamin D could mediate diverse action throughout the cardiovascular system. Alsharairi summarized in his commentary that adipose tissue is the most important reservoir for 25(OH)D and can activate/deactivate the bioavailability of vitamin D through specific hydroxylation processes.^13^ Low 25(OH)D concentration in obese individuals may contribute to inflammatory by increasing macrophage and monocyte cell surface expression of Toll-line receptors (TLR-2, TLR-4).^39^

We found a negative association between 25(OH)D and SBP (r=−0.035, p=0.007) in Chinese children after adjusting various potential confounders. The sex-stratified analysis revealed that the risk of hypertension was not significantly different across vitamin D status groups in boys but was significantly higher in vitamin D insufficient subgroup among girls. Girls versus boys were proven to have more fat mass, which can decrease bioavailability of vitamin D due to excess storage in it. Additionally, a cross-sectional study found that expression of the 1α-hydroxylases CYP27B and CYP2J2 was reduced only in the subcutaneous adipose tissue of obese women.^40^ Therefore, the differences in fat distribution between sexes may account for the gender-specific associations. Vitamin D may regulate BP through the renin-angiotensin-aldosterone system, which is supported by the evidence that the VDR knockout mice experienced elevated renin and BP.^41^ Furthermore, chronic vitamin D deficiency causes secondary hyperparathyroidism, which may mediate many cardiometabolic risk factors in turn.^3^

Individuals with insufficient vitamin D in our study had 1.47 times (95% CI 1.26 to 1.70) of risk for hyperglycemia compared with those with adequate vitamin D. A cohort study^42^ followed for 31 years in Finland found that children who regularly took 2000 IU daily of vitamin D_3_ during first year of life had an approximately 80% reduced risk of type 1 diabetes. Additionally, we found vitamin D insufficiency had an additive interaction with excess weight on glycemic metabolism. The proportion of joint effect on hyperglycemia due to interaction was 32% (95% CI 0.14% to 0.51%). There is evidence that vitamin D may directly stimulate pancreatic insulin secretion through nuclear VDR found in the pancreatic islet β-cells.^43^ The bioavailability of vitamin D can be decreased by the excess storage in adipose tissue, resulting in an increased risk of adverse glycemic outcomes.^44^

Ganji *et al* 5 reported that HOMA-IR was inversely related to serum 25(OH)D in US children using data released by the National Center for Health Statistics. Likewise, our present study observed negative associations between 25(OH)D and insulin and HOMA-IR in Chinese children. However, the risk of insulin resistance in individuals with insufficient vitamin D was not significantly higher than those with adequate vitamin D in our study, especially in the normal weight children (OR: 1.06, 95% CI 0.87 to 1.29). Moreover, a randomized placebo-controlled trial found vitamin D supplementation had no effects on insulin sensitivity or secretion in vitamin D deficient, overweight or obese adults.^45^ These inconsistent results may be partly explained by small sample size to obtain enough efficacy, and residual confounding of unmeasured or uncontrolled confounders. And the non-significant association between insufficient vitamin D and insulin resistance may result from adjusting for the body composition.

A growing number of epidemiological evidence^21 46 47^ indicate the levels of fat-soluble vitamin D are inversely associated with atherogenic lipid profiles. Our study extended prior investigations and first showed inversely U-shaped associations between lipid profiles (TC, LDL-C, and HDL-C) and plasma 25(OH)D concentrations. Here, we found vitamin D insufficiency was associated with abnormal TC only in girls. The gender difference may be due to reduced intake of vitamin D and the lack of outdoor activity.^9^ A Mendelian randomization study^48^ has provided the evidence that single nucleotide polymorphisms causing variations in 25(OH)D can lead to a genetically increased remnant lipoprotein-cholesterol.

Observational studies strongly associated vitamin D insufficiency with a various range of cardiovascular disease risk factors beyond defects in bone and calcium metabolism.^5 6 8 13 21 49^ However, studies evaluating vitamin D supplementation or treatment on cardiovascular health have not drawn a certain conclusion on its beneficial effects. Recently, Scragg *et al* 50 randomized 5108 adults from the general population to monthly, high-dose oral vitamin D or placebo for a median of 3.3 years. They did not observe any significant differences in the combined endpoint of incident cardiovascular disease or death between groups. However, several points need to be considered when interpreting the results of vitamin D supplementation trials. Participants, whose vitamin D levels lie in the range where disease risk changes with vitamin D status, should be eligible in the trials because of the non-linear associations between vitamin D status and diseases.^51^ Additionally, low response rates and outcomes that are rare or long term can make the trials unlikely to give a clear answer about benefits of the vitamin D supplementation.

Previous studies have reported that Chinese children aged 12–14 years had the highest prevalence of vitamin D insufficiency, which may result in changes of body composition during pubertal maturation and longtime indoor learning.^52^ Thus, widespread use of vitamin D supplements should be recommended to this age group. This study had several strengths, including large sample size, school-based sample of children, rigorous quality control procedures, Vitamin D Standardization-Certification Program certified assay, and adjustment of diverse potential confounders. This study has several limitations. First, information on sun exposure was not available in our study; however, we adjusted geographical location and PA in the analysis, which to some extent can reflect sun exposure. Second, the nature of this cross-sectional study limits a causal inference between vitamin D insufficiency and cardiometabolic risk factors. Third, although our data were collected in a large-scale pediatric sample, the generalizability may be limited due to a lack of other ethnic groups. Fourth, data on vitamin D supplementation were not available in our survey, but dietary vitamin D intake information was used to control for potential confounding effect.

In conclusion, this cross-sectional survey with a large sample size of Chinese children showed that children with inadequate 25(OH)D concentrations are at higher risk of various cardiometabolic risk factors. Prospective studies are warranted to establish whether there is a causal association between vitamin D levels and cardiometabolic risk in children. Moreover, we found that 32% of the risk of hyperglycemia can be explained by the interaction between vitamin D insufficiency and high BMI, which suggested that effective sun exposure and vitamin D supplementation should be encouraged in school-age children for the prevention of further cardiometabolic risk, especially in the obese children. The non-linear associations between vitamin D status and cardiometabolic parameters observed in this study indicated that dose–response relationship should be taken into consideration when analyzing the effect of vitamin D on the prevention of cardiometabolic risk in further vitamin D supplementation trails.
