# Supplementary material for: Adequate 25-hydroxyvitamin D levels are inversely associated with various cardiometabolic risk factors in Chinese children, especially obese children
Source: BMJ Open Diabetes Res Care. 2020 Feb 17;8(1):e000846. doi: 10.1136/bmjdrc-2019-000846 (PMC7039595; doi:10.1136/bmjdrc-2019-000846)
Supplement: Supplementary data [file bmjdrc-2019-000846supp001.pdf]

SUPPLEMENTAL MATERIAL

Adequate 25-hydroxyvitamin D levels are Inversely Associated with Various Cardiometabolic Risk Factors in Chinese Children, especially Obese Children

Supplemental Table 1. Sensitivity analysis: partial correlations between 25(OH)D and cardiometabolic parameters

Supplemental Figure 1. Nonlinear associations of 25(OH)D with cardiometabolic parameters in Chinese children and adolescents

Supplemental Figure 2. Multivariate-adjusted means or geometric means of cardiometabolic parameters across different vitamin D status groups in boys

Supplemental Figure 3. Multivariate-adjusted means or geometric means of cardiometabolic parameters across different vitamin D status groups in girls

Supplemental Table 2. Multivariate-adjusted odds ratios (ORs) and 95% CIs of cardiometabolic risk factors according to vitamin D and abdominal obesity status

Supplemental Table 1. Sensitivity analysis: partial correlations between 25(OH)D and cardiometabolic parameters *

Cardiometabolic parameters	All		Boys		Girls	
	r	***p* value**	***r***	***p* value**	***r***	***p* value**
WHtR	0.040	0.002	0.053	0.003	0.029	0.117
SBP	-0.035	0.007	0.022	0.230	-0.107	<0.001
DBP	-0.011	0.383	0.040	0.029	-0.068	<0.001
TC	0.007	0.559	0.001	0.945	0.012	0.504
LDL-C	0.011	0.384	0.001	0.949	0.018	0.329
HDL-C	0.043	<0.001	0.051	0.005	0.035	0.054
TG †	-0.043	<0.001	-0.043	0.017	-0.041	0.024
FBG †	-0.035	0.006	-0.028	0.022	-0.040	0.026
Insulin †	-0.063	<0.001	-0.065	<0.001	-0.064	<0.001
HOMA-IR †	-0.070	<0.001	-0.069	0.001	-0.072	<0.001

Abbreviations: WHtR, Waist to height ratio; SBP, systolic blood pressure; DBP, diastolic blood pressure; TC, total cholesterol; LDL-C, low density lipoprotein-cholesterol; HDL-C, high density lipoprotein-cholesterol; TG, triglyceride; FBG, fasting blood glucose; HOMA-IR, Homeostasis model assessment-insulin resistance.

* Model adjusted for age, sex (not for adjusted in stratified analysis), season of blood collection, geographical location, smoking, drinking, physical activity, dietary vitamin D intake, BMI, fat mass index, and MMI.

† Log-transformed.

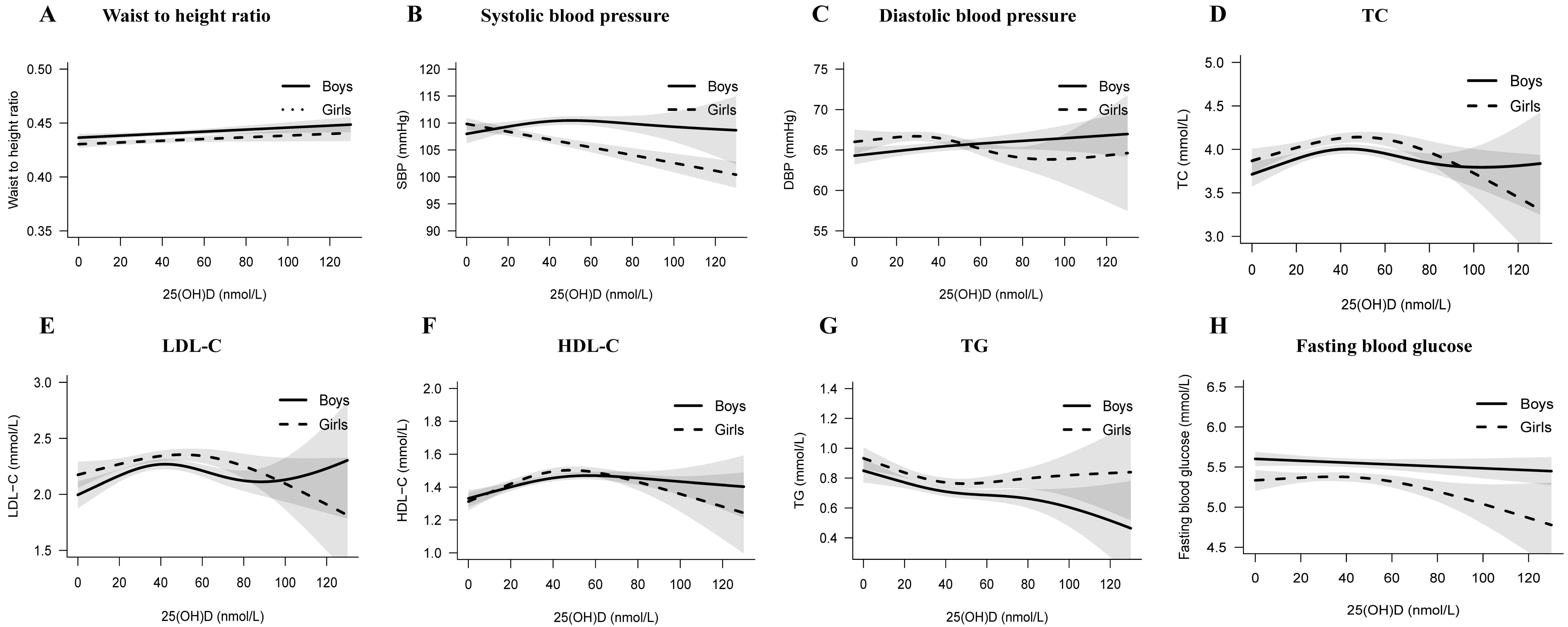

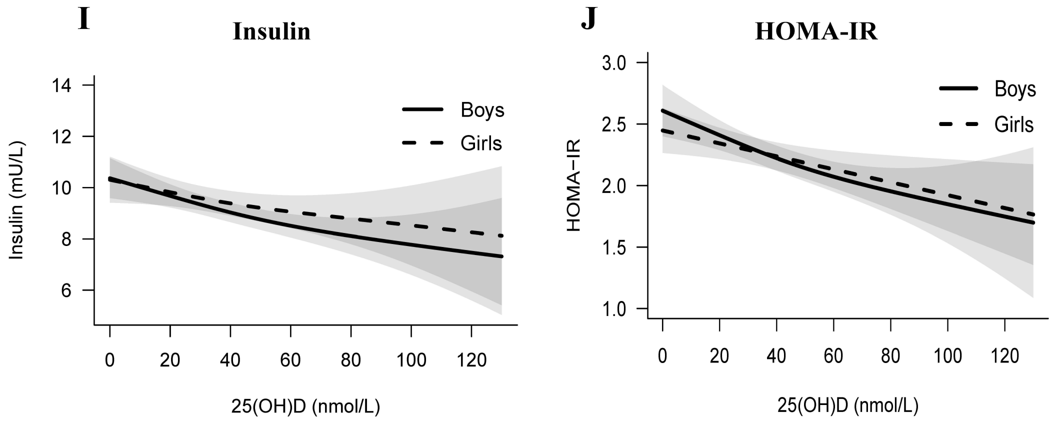

Supplemental Figure 1. Nonlinear associations of 25(OH)D with cardiometabolic parameters in Chinese children and adolescents. (A), (B), (C), (D), (E), (F), (G), (H), (I), and (J) stand for the adjusted means of WhtR, SBP, DBP, TC, LDL-C, HDL-C, TG, FBG, insulin, and HOMA-IR, respectively. Means are adjusted for age, season of blood collection, geographical location, smoking, drinking, physical activity, dietary vitamin D intake, BMI, FMP, and MMI. Solid line denotes boys, dotted line denotes girls, and shading indicates 95% CI.

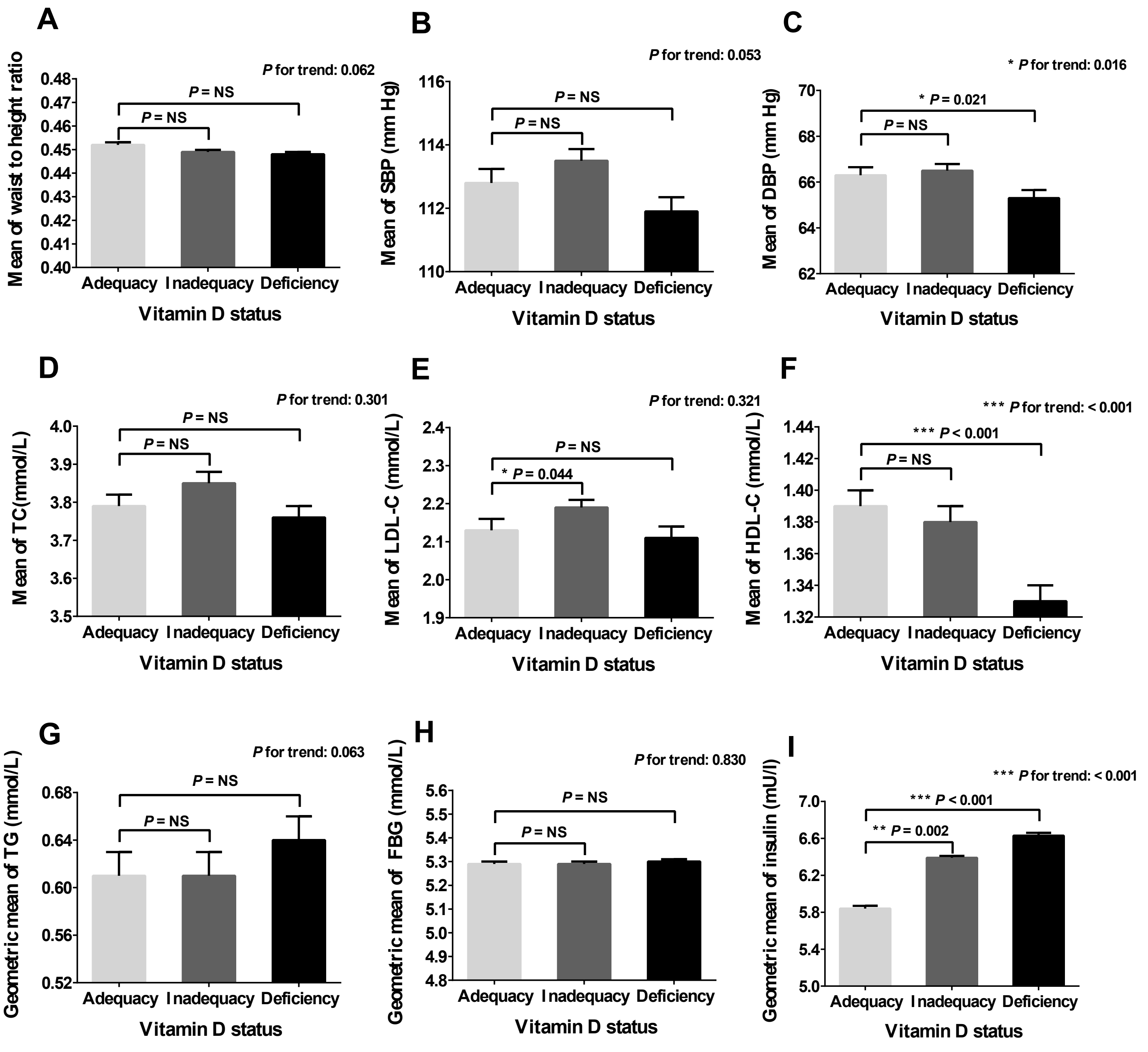

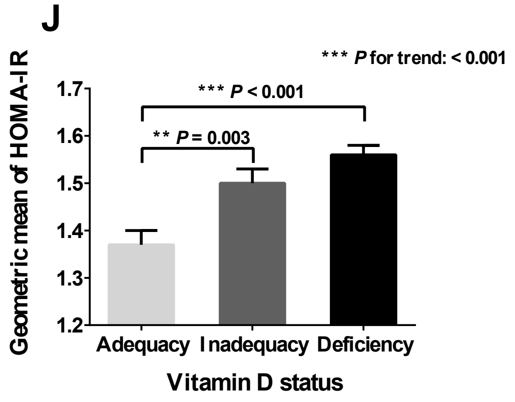

Supplemental Figure 2. Multivariate-adjusted means or geometric means of cardiometabolic parameters across different vitamin D status groups in boys. (A), (B), (C), (D), (E), (F), (G), (H), (I), and (J) stand for the adjusted means (or geometric means) of waist to height ratio, SBP, DBP, TC, LDL-C, HDL-C, TG, FBG, insulin, and HOMA-IR, respectively. Means (or geometric means) are adjusted for age, season of blood collection, geographical location, smoking, drinking, physical activity, dietary vitamin D intake, BMI, FMP, and MMI. Error bars stand for standard error. * 0.01≤P<0.05, ** 0.001≤P<0.01, *** P<0.001.

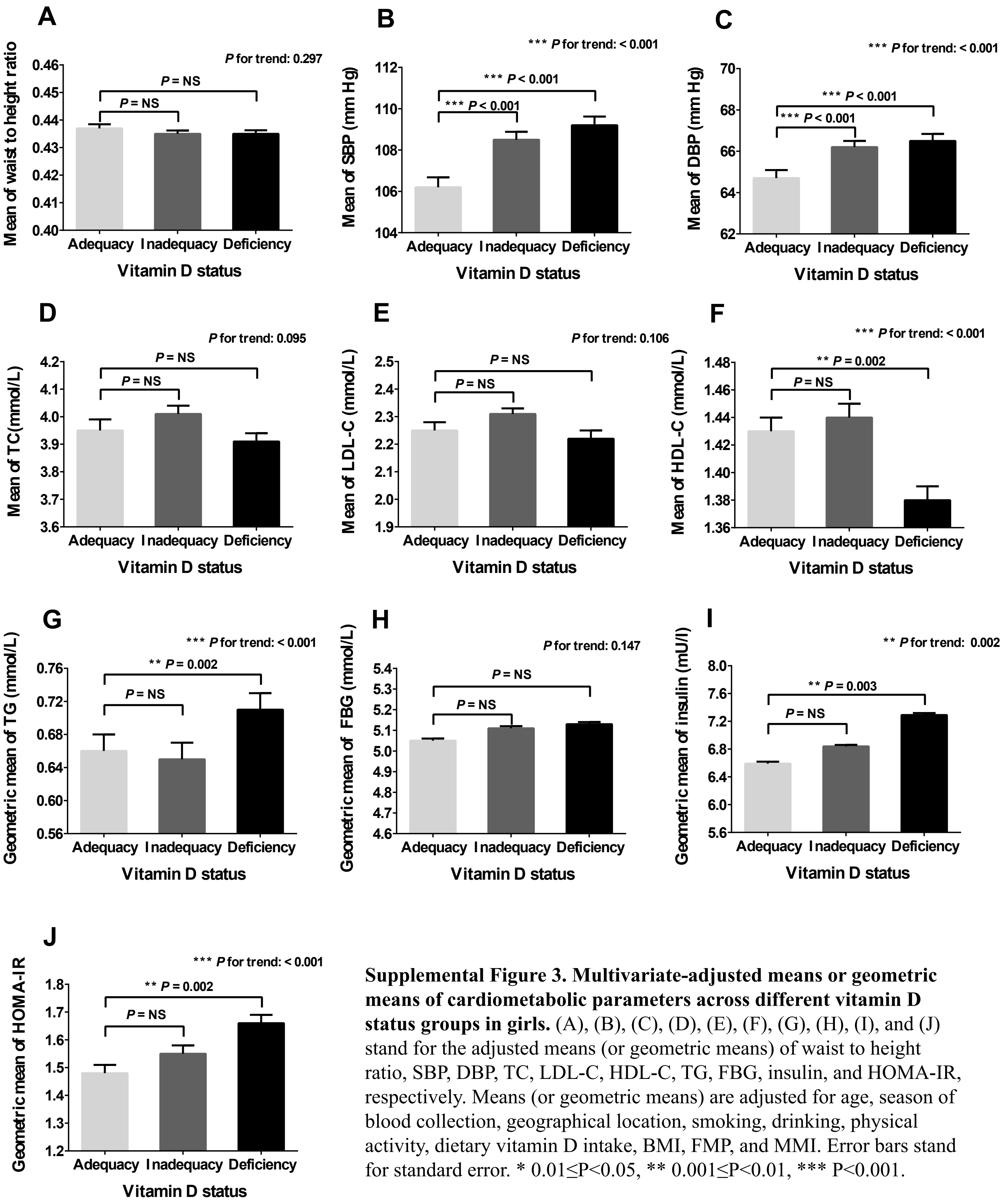

Supplemental Figure 3. Multivariate-adjusted means or geometric means of cardiometabolic parameters across different vitamin D status groups in girls. (A), (B), (C), (D), (E), (F), (G), (H), (I), and (J) stand for the adjusted means (or geometric means) of waist to height ratio, SBP, DBP, TC, LDL-C, HDL-C, TG, FBG, insulin, and HOMA-IR, respectively. Means (or geometric means) are adjusted for age, season of blood collection, geographical location, smoking, drinking, physical activity, dietary vitamin D intake, BMI, FMP, and MMI. Error bars stand for standard error. * 0.01≤P<0.05, ** 0.001≤P<0.01, *** P<0.001.

Supplemental Table 2. Multivariate-adjusted odds ratios (ORs) and 95% CIs of cardiometabolic risk factors according to vitamin D and abdominal obesity status [*]

Cardiometabolic risk factors [†]	Abdominal Obesity [‡]	Vitamin D status [§]	OR (95% CI)	*p* for interaction [ǁ]	Measures of additive interaction	
					RERI (95% CI)	AP (95% CI)
Hypertension	Non-abdominal obesity	Adequacy	1.00	0.290	-0.26 (-0.87-0.33)	-0.19 (-0.62 - 0.24)
		Insufficiency	1.16 (0.97 - 1.40)			
	Abdominal obesity	Adequacy	1.50 (1.00 - 2.26) [¶]			
		Insufficiency	1.40 (1.06 - 1.85) [¶]			
High TC	Non-abdominal obesity	Adequacy	1.00	0.811	0.63 (-1.04 - 2.31)	0.23 (-0.36 - 0.82)
		Insufficiency	1.58 (0.97 - 2.57)			
	Abdominal obesity	Adequacy	1.52 (0.53 - 4.35)			
		Insufficiency	2.73 (1.43 - 5.21) [¶]			
High LDL-C	Non-abdominal obesity	Adequacy	1.00	0.922	0.21 (-1.19 - 1.62)	0.09 (-0.51 - 0.69)
		Insufficiency	1.54 (0.94 - 2.51)			
	Abdominal obesity	Adequacy	1.59 (0.65 - 3.94)			
		Insufficiency	2.34 (1.24 - 4.42) [¶]			
Low HDL-C	Non-abdominal obesity	Adequacy	1.00	0.676	-0.05 (-0.92 - 0.81)	-0.03 (-0.49 - 0.43)
		Insufficiency	1.28 (0.95 - 1.73)			
	Abdominal obesity	Adequacy	1.65 (0.94 - 2.89)			
		Insufficiency	1.88 (1.21 - 2.91) [¶]			
High TG	Non-abdominal obesity	Adequacy	1.00	0.298	-0.24 (-1.84 -1.35)	-0.08 (-0.58 - 0.43)
		Insufficiency	1.78 (1.05 - 3.03) [¶]			
	Abdominal obesity	Adequacy	2.63 (1.25 - 5.55) [¶]			
		Insufficiency	3.17 (1.70 - 5.91) [¶]			

Hyperglycemia	Non-abdominal obesity	Adequacy	1.00	0.121	0.17 (-0.10 - 0.44)	0.20 (-0.11 - 0.51)
		Insufficiency	1.14 (0.98 - 1.33)			
	Abdominal obesity	Adequacy	0.55 (0.37 - 0.81) ¶			
		Insufficiency	0.86 (0.67 - 1.11)			
Insulin Resistance	Non-abdominal obesity	Adequacy	1.00	0.465	-0.16 (-0.63 - 0.31)	-0.14 (-0.55 - 0.27)
		Insufficiency	1.17 (0.98 - 1.40)			
	Abdominal obesity	Adequacy	1.16 (0.78 - 1.72)			
		Insufficiency	1.17 (0.89 - 1.54)			

Abbreviations: TC, total cholesterol; TG, triglyceride.

* The analysis was adjusted for age, sex, season of blood collection, geographical location, smoking, drinking, physical activity, dietary behaviors, BMI, FMP, and MMI.

† The diagnostic criteria are as follows:

Hypertension was classified by the 95th sex-, age- and height-specific blood pressure cutpoints of Chinese standard;[16]

Abnormal blood lipids levels were classified by the age- and sex-specific lipoprotein cutpoints of Chinese children;[17]

Hyperglycemia was defined as fasting blood glucose ≥5.6 mmol/L;[18]

Insulin Resistance was defined by the World Health Organization as values in the highest quartile of the HOMA-IR.[19]

‡ Abdominal obesity was defined as waist to height ratio ≥0.5.[15]

§ The vitamin D status was classified as sufficiency (≥50 nmol/L) and insufficiency (<50 nmol/L) according to the IOM recommendation.[20]

‖ A cross-product interaction term was included in the logistic regression model to assess multiplicative interaction.

¶ Significantly different from the referent category, vitamin D sufficiency and non-abdominal obesity group.
